# A phase II study of ramucirumab and somatostatin analog therapy in patients with advanced neuroendocrine tumors

**DOI:** 10.1093/oncolo/oyae364

**Published:** 2025-01-21

**Authors:** Kimberly Perez, Matthew H Kulke, Hui Zheng, Jill Allen, Jeffrey Clark, Andrea C Enzinger, Peter C Enzinger, Bruce E Johnson, Nadine J McCleary, Aparna Parikh, Anuj Patel, Douglas Rubinson, Matthew B Yurgelun, Kaitlyn Ramsey, Emma Johnson, Christopher Graham, Jennifer A Chan

**Affiliations:** Department of Medical Oncology, Dana-Farber Cancer Institute, Boston, MA 02215, United States; Harvard Medical School, Boston, MA 02115, United States; Section of Hematology and Oncology, Boston University and Boston Medical Center, Boston, MA 02118, United States; Biostatistics Center, Massachusetts General Hospital, Boston, MA 02114, United States; Harvard Medical School, Boston, MA 02115, United States; Department of Medicine, Massachusetts General Hospital, Boston, MA 02114, United States; Harvard Medical School, Boston, MA 02115, United States; Department of Medicine, Massachusetts General Hospital, Boston, MA 02114, United States; Department of Medical Oncology, Dana-Farber Cancer Institute, Boston, MA 02215, United States; Harvard Medical School, Boston, MA 02115, United States; Department of Medical Oncology, Dana-Farber Cancer Institute, Boston, MA 02215, United States; Harvard Medical School, Boston, MA 02115, United States; Department of Medical Oncology, Dana-Farber Cancer Institute, Boston, MA 02215, United States; Harvard Medical School, Boston, MA 02115, United States; Department of Medical Oncology, Dana-Farber Cancer Institute, Boston, MA 02215, United States; Harvard Medical School, Boston, MA 02115, United States; Harvard Medical School, Boston, MA 02115, United States; Department of Medicine, Massachusetts General Hospital, Boston, MA 02114, United States; Department of Medical Oncology, Dana-Farber Cancer Institute, Boston, MA 02215, United States; Harvard Medical School, Boston, MA 02115, United States; Department of Medical Oncology, Dana-Farber Cancer Institute, Boston, MA 02215, United States; Harvard Medical School, Boston, MA 02115, United States; Department of Medical Oncology, Dana-Farber Cancer Institute, Boston, MA 02215, United States; Harvard Medical School, Boston, MA 02115, United States; Department of Medical Oncology, Dana-Farber Cancer Institute, Boston, MA 02215, United States; Department of Medical Oncology, Dana-Farber Cancer Institute, Boston, MA 02215, United States; Department of Medical Oncology, Dana-Farber Cancer Institute, Boston, MA 02215, United States; Department of Medical Oncology, Dana-Farber Cancer Institute, Boston, MA 02215, United States; Harvard Medical School, Boston, MA 02115, United States

**Keywords:** ramucirumab, extrapancreatic neuroendocrine tumors, carcinoid tumors

## Abstract

**Objectives:**

Well-differentiated neuroendocrine tumors (NET) are highly vascular tumors characterized by their expression of vascular endothelial growth factor (VEGF). This trial investigated the activity of ramucirumab, a monoclonal antibody that targets VEGF receptor-2 (VEGFR-2) and inhibits activity of VEGF, in combination with somatostatin analog therapy in patients (pts) with advanced extra-pancreatic NET.

**Methods:**

We conducted a single-arm phase II trial enrolling pts with advanced, progressive extra-pancreatic NET. Patients were treated with ramucirumab 8 mg/kg intravenously on days 1 and 15 of each 28-day cycle. The primary endpoint was progression-free survival (PFS). Secondary endpoints toxicity, radiographic and biochemical tumor response rate, and overall survival (OS).

**Results:**

The trial enrolled 43 patients. Primary tumor sites included small intestine 20 (46%), lung 10 (23%), thymus 3 (7%), rectum 1(2%), kidney 1(2%), and unknown primary 8(18%). Median PFS was 14.2 months (95% CI, 9.0-25.6 months), and median OS was 24.9 months (95% CI, 20.7-43.1 months). Best response by RECIST 1.1: partial response 5% (95% CI, 0.6%-15.8%). Chromogranin A levels dropped by at least 50% in 10% of the 37 patients who had elevated levels at baseline. Most common all-grade adverse events included fatigue (84%) and hypertension (84%).

**Conclusion:**

Ramucirumab demonstrated efficacy and safety in this single-arm phase II trial. These findings support the continued evaluation of angiogenesis inhibitors in the treatment of NET.

**Clinical trial registration:**

NCT02795858.

**Implications for practice**

Targeting of proangiogenic pathways by tyrosine kinase receptors has resulted in positive impacts on PFS in patients with well-differentiated NETs. Evaluation of monoclonal antibody VEGF/VEGFR inhibitors has resulted in mixed outcomes and toxicity profiles that have limited expanded use. In this phase II trial, the median PFS observed was like that reported in prospective trials which evaluated the role of bevacizumab and ziv-aflibercept in extra-pancreatic NETs. The most common toxicity that results with angiogenesis inhibition is hypertension, but the incidence seems to vary with each agent. In this trial any grade hypertension was most common in patients with baseline hypertension. Treatment with ramucirumab was associated with encouraging efficacy and acceptable toxicity in patients with advanced, progressive extra-pancreatic NET. These results support continued investigation of ramucirumab in the treatment of NET.

## Introduction

Neuroendocrine neoplasms originate from the neuroendocrine cells throughout the body, with a phenotype that can be quite heterogeneous. Well-differentiated neuroendocrine tumors (NETs) are highly vascularized neoplasms characterized by expression of vascular endothelial growth factor-A (VEGF-A). VEGF-A binds to the vascular endothelial growth factor receptors (VEGFR)-1 and -2 and induces endothelial cell proliferation and angiogenesis.^1^

Advances in treatment for well-differentiated neuroendocrine tumors over the last decade have translated into improved survival and patient outcomes. In addition to somatostatin analogs, the treatment landscape now includes everolimus, Lu-177 dotatate, and several multi-targeted tyrosine kinase inhibitors (TKIs) targeting VEGFR. In randomized placebo-controlled trials, pazopanib, surufatinib, and cabozantinib have improved progression-free survival in patients with extra-pancreatic NET.^2–6^ Additionally, bevacizumab and Ziv-aflibercept, monoclonal antibodies targeting VEGF, have demonstrated similar single-agent activity in phase II and III trials enrolling patients with advanced extra-pancreatic NET.^7,8^

Ramucirumab is a human receptor-targeted monoclonal antibody that specifically binds VEGFR-2 which prevents interaction with activating ligands (VEGF-A, VEGF-C, and VEGF-D). Binding results in inhibition of ligand-stimulated activation of VEGFR-2 and associated downstream intracellular signaling components, including Erk1/Erk2, thereby neutralizing ligand-induced proliferation and migration of human endothelial cells.^9,10^

Based on the efficacy of VEGF pathway inhibitors in NET, we conducted a phase II trial to determine the clinical efficacy and safety profile of ramucirumab in patients with advanced extra-pancreatic NET.

## Materials and methods

### Study design and participants

We conducted a single-arm phase II trial that enrolled patients with histologically confirmed metastatic or locally advanced well- or moderately differentiated neuroendocrine tumors of extra-pancreatic origin not amenable to curative resection. Participants were required to have evidence of disease progression within 12 months prior to study entry; progressive disease by Response Evaluation Criteria in Solid Tumors [RECIST] criteria were not required. Patients were further required to have measurable disease by RECIST 1.1, Eastern Cooperative Oncology Group (ECOG) performance status of ≤1; adequate hepatic function (serum bilirubin ≤1.5 times upper limit of normal (ULN); aspartate transaminase (AST) ≤2.5 times ULN (≤5 times if liver metastases were present)); and adequate bone marrow function (absolute neutrophil count ≥ 1500/mm^3^; platelets ≥ 100 000/mm^3^). There was no limit to prior treatments. Prior therapy with anti-VEGF therapy was permitted unless it was discontinued due to unacceptable toxicity.

All patients provided signed, informed consent as required by the institutional review boards of the participating centers, which included Dana-Farber Cancer Institute and Massachusetts General Hospital.

### Study treatment

Patients received ramucirumab (Eli Lily and Company) at a dose of 8 mg/kg intravenously on days 1 and 15 of each 28-day treatment cycle. Somatostatin analog therapy with octreotide long-acting release depot or lanreotide was administered concurrently with ramucirumab throughout the protocol. Somatostatin analog-naïve patients were allowed to initiate treatment during the screening period prior to the start of ramucirumab. Patients were continued on ramucirumab and somatostatin analog treatment until progression, unacceptable toxicity, or withdrawal of consent. Specific adverse events mandating discontinuation of protocol therapy included any grade of gastrointestinal perforation or fistula, congestive heart failure, reversible posterior leukoencephalopathy, grades 3 or 4 hemorrhage or arterial thromboembolic event, recurrent grade 3 or 4 venous thromboembolic event, recurrent grade 3 or any occurrence of grade 4 hypertension.

Pre-treatment and on-study assessments included history, physical examination, and laboratory tests including complete blood count, electrolytes, and liver function tests. Disease was assessed with restaging scans after every 3 cycles of treatment. Tumor markers, serum chromogranin A, and 24-hour urine 5-HIAA were measured at baseline and again at each radiographic restaging if elevated on baseline measurement.

### Outcomes

The primary endpoint was progression-free survival (PFS). Secondary endpoints included toxicity, overall survival (OS), objective response rate by RECIST 1.1, and biochemical response utilizing serum chromogranin and 24-hour urine 5-HIAA.

### Statistical analysis

progression-free survival (PFS) was defined from the date of enrollment to the date of documented progression or death from any cause. With a planned sample size of 43 patients, we anticipated detecting a difference between the null hypothesis median PFS of 8 months and the alternative hypothesis median PFS of 12 months with a power of 80% and one-sided alpha of 0.05. Overall survival (OS) was defined as the time between study enrollment and death due to any cause. Distributions of PFS and OS were estimated using the Kaplan-Meier method, and the 95% CI was estimated using the Greenwood formula.

Secondary objectives included toxicity of ramucirumab in combination with somatostatin analog therapy, as defined by the Common Terminology Criteria for Adverse Events version 4.0. A safety analysis was planned after the first 10 patients completed one cycle of therapy; enrollment was to be expanded if no patients experienced toxicity requiring discontinuation of therapy.

An exploratory objective included an assessment of biochemical response in patients with elevated chromogranin A at baseline. Levels of chromogranin-A were measured at baseline and following treatment with ramucirumab. The biochemical response was defined as a greater than 50% drop in chromogranin A from baseline.

SAS version 9.4 (SAS Institute, Inc.) was used for data analyses.

## Results

### Patient characteristics

Forty-three patients with advanced extra-pancreatic NET were enrolled between July 1, 2016, and December 31, 2022. Patients had a median age of 64 years (range 36-77 years); 24 (56%) were male and 18 (42%) had ECOG performance status of 0. The most common primary tumor sites included small intestine (*n* = 20; 46%), lung (*n* = 10; 23%), and unknown primary (*n* = 8; 18%). Most patients had tumors with pathologic grades of 1 or 2. Thirty-five (81%) were taking a somatostatin analog prior to starting the trial. Prior therapies included everolimus (*n* = 24; 56%) cytotoxic chemotherapy (*n* = 13; 30%), other VEGF pathway inhibitors (*n* = 11; 26%), radiation therapy (*n* = 12; 28%), or peptide receptor radionuclide therapy (*n* = 3; 7%) (see Table 1).

**Table 1.** Demographics.

Number of patients	43
Median age at diagnosis, years (range)	64 (36 to 77)
Sex, *N* (%)	
Male	24 (56)
Female	19 (44)
*Race, N (%)*	
Asian	1 (2.5)
Black or African American	1 (2.5)
White	41 (95)
*ECOG, N (%)*	
0	18 (42)
1	23 (53)
Not available	2 (5)
*Baseline hypertension, N (%)*	
Yes	21 (49)
No	22 (51)
Patients with increased baseline chromogranin A (>95 U/mL)	37
*Baseline chromogranin A, ng/mL*	
Median	522
Range	28-12 860
*Histologic grade of extra-pulmonary/thymic NET cohort, N (%)*	
Low (grade 1)	8 (18)
Intermediate (grade 2)	7 (16)
High (grade 3)	3 (7)
Unknown	12 (28)
*Histologic variants of pulmonary/thymic cohort, N (%)*	
Typical	1 (2)
Atypical	10 (23)
Large cell	1 (2)
Unknown	2 (4)
*Primary tumor location, N (%)*	
Small intestine	20 (46)
Lung	10 (23)
Thymus	3 (7)
Rectum	1 (2)
Kidney	1 (2)
Unknown	8 (18)
*Prior therapy, N (%)*	
Somatostatin analog	35 (81%)
Chemotherapy	13 (30)
Interferon	1 (2)
Everolimus	24 (56)
*VEGF pathway inhibitor*	
Cabozantinib	5 (12)
Axitinib	1 (2)
Sunitinib	1 (2)
Ziv-Aflibercept	3 (7)
Sorafenib	1 (2)
Investigational therapy^*^	3 (7)
Liver directed therapy	1 (2)
Radiation therapy	12 (28)
Peptide radionuclide receptor therapy	3 (7)

^*^Investigational therapies included pembrolizumab, 2-,methoxyestradiol, VB-111(ofranergene obadenovec).

**Figure 1.**
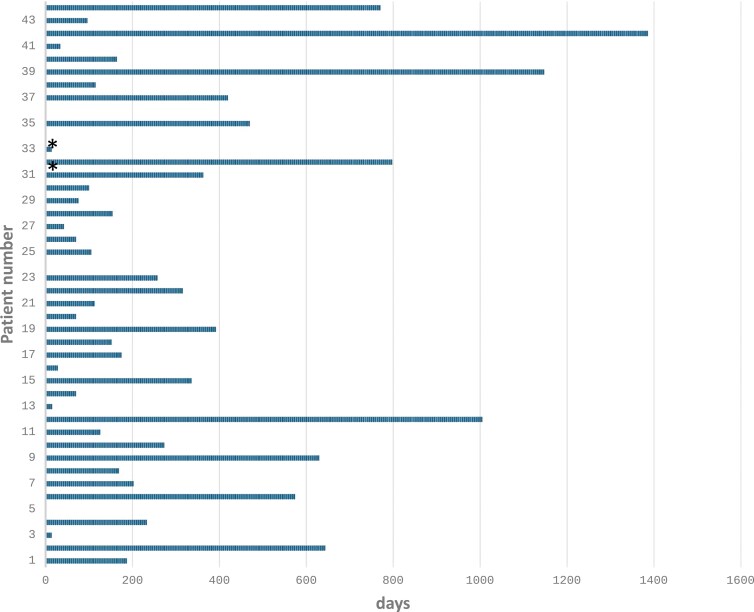
Treatment duration.

Patients completed a median of 6 cycles of therapy (range, 0-44 cycles) (see Figure 1). The median follow-up time was 23.5 months (range: 1.7-76.6 months). Reasons for treatment discontinuation included disease progression 19 (44%), unacceptable toxicity 5 (12%), prolonged treatment break 4 (9%), withdrawal of consent 4 (9%), clinician discretion 7(16%), and death 4 (9%).

### Efficacy

**Figure 2.**
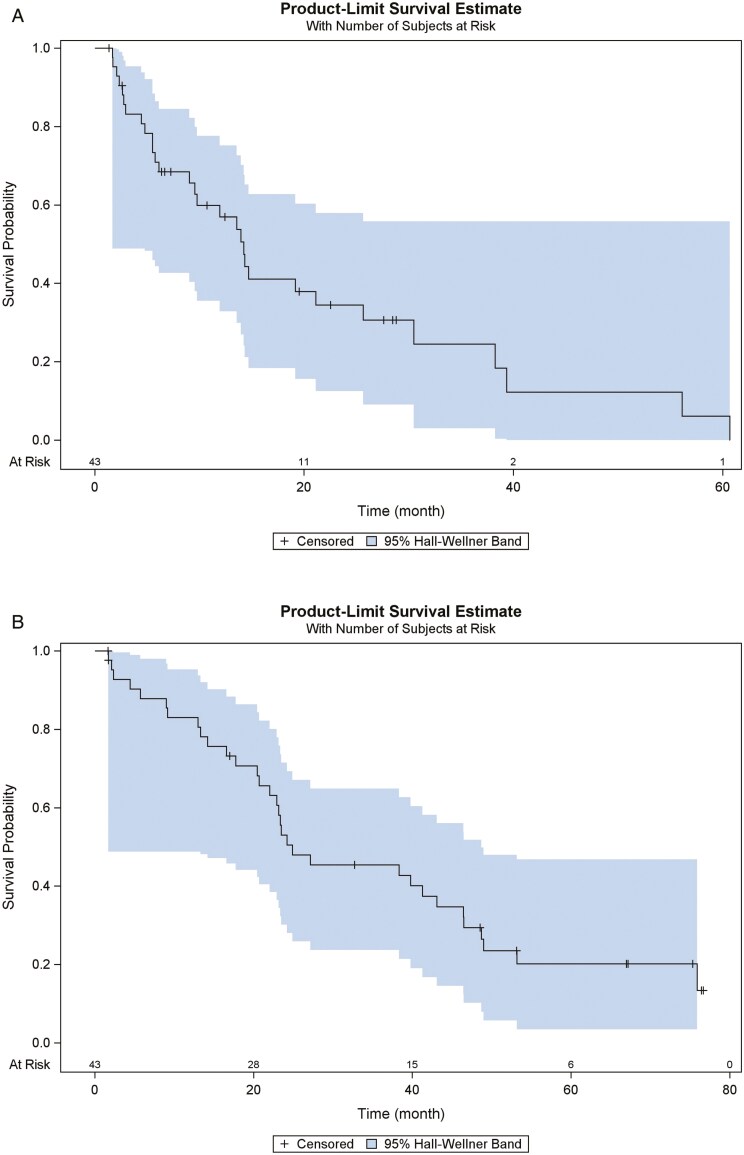
(A) Progression free survival (PFS) and (B) Overall survival (OS).

The median PFS duration was 14.2 months (95% CI, 9.0-25.6 months). Median OS duration was 24.9 months (95% CI, 20.7-43.1 months). All 43 patients were evaluated for objective tumor response by RECIST 1.1 criteria. Partial response was observed in 2 patients (5%; 95% CI, 0.6%-15.8%). The best response of stable disease was observed in 33 patients (77%, 95% CI, 61.4%-88.2%) and progressive disease in 3 patients (7%; 95% CI, 1.5%-19.1%). Five patients did not undergo restaging due to early withdrawal (see Figure 2A and B).

Baseline serum chromogranin A was elevated in 37 patients. Four of these patients (10%) patients had a drop greater than 50% from baseline. Assessment of biochemical response in 24-hour urine 5-HIAA was not possible due to inadequate follow-up results.

### Toxicity

All 43 patients treated were evaluated for toxicity, as summarized in Table 2. All 43 patients experience at least one treatment-related adverse event. Hypertension of any grade was reported in 36 patients (84%), with 15 patients (35%) experiencing grade 3 hypertension while receiving ramucirumab. Twenty-one patients had a diagnosis of hypertension prior to beginning treatment with ramucirumab; notably, 5 of the 15 patients who developed grade 3 hypertension did not have hypertension at baseline. Other grade 3 treatment-related toxicities included proteinuria (*n* = 2; 5%), elevated aspartate aminotransferase (*n* = 2; 5%), headache, diarrhea, elevated alanine aminotransferase, hyponatremia, hypokalemia, dyspnea, and syncope (each *n* = 1; 2%). No treatment-related deaths were reported.

**Table 2.** Toxicities by CTCAE version 4.0.

Category	Grade II, *N* (%)	Grade III, *N* (%)
Hypertension	13 (30)	15 (35)
Sinus bradycardia	1 (2)	
Chest pain	1 (2)	
Thromboembolic event	1 (2)	
Elevated creatinine	4 (9)	
Renal insufficiency NOS	1 (2)	
Proteinuria	11 (25)	2 (5)
Hematuria	1 (2)	
Thrombocytopenia	3 (7)	
Anemia	4 (9)	
Low WBC	3 (7)	
Neutropenia	2 (5)	
Diarrhea	2 (5)	1 (2)
Nausea	2 (5)	
Elevated alkaline phosphatase	1 (2)	
Elevated aspartate aminotransferase	1 (2)	2 (5)
Elevated alanine aminotransferase	1 (2)	1 (2)
Hyperbilirubinemia	1 (2)	
Hyponatremia		1 (2)
Hypokalemia		1 (2)
Hypomagnesemia	1 (2)	
Hypothyroidism	1 (2)	
Dyspnea		1 (2)
Syncope		1 (2)
Headache	1 (2)	1 (2)
Dizziness	1 (2)	
Skin changes	1 (2)	
Lower extremity edema	5 (12)	
Fever	1 (2)	
Fatigue	5 (12)	
Anorexia	2 (5)	
Dehydration	1 (2)	
Generalized weakness	1 (2)	
Weight loss	1 (2)	
Joint/muscle pain	1 (2)	

## Discussion

Well-differentiated NETs are highly vascularized malignancies arising from cells of the diffuse neuroendocrine system. VEGF is constitutively expressed by normal neuroendocrine cells and retained in upwards of 80% of NETs. The VEGF pathway plays a key role in angiogenesis impacting tumor biology and progression.^11^ Proangiogenic pathways can be blocked at different levels, including direct suppression of proangiogenic molecules including VEGF, or inhibition of tyrosine kinase receptors such as VEGFR. VEGF inhibitors, including bevacizumab, and tyrosine kinase inhibitors targeting VEGFR have demonstrated activity against NET and encouraging impacts on PFS.^6,12,13^ In this phase II trial, we observed a median PFS of 14.2 months in patients with extra-pancreatic NET treated with ramucirumab, a monoclonal antibody targeting VEGFR-2. Although the trial was a single-arm uncontrolled study, the PFS duration that we observed is like what has been described with other anti-VEGF pathway inhibitors. Specifically, the median PFS was 16.6 months in patients with extra-pancreatic NET treated with bevacizumab in the phase III SWOG S0518 trial, and a median PFS of 11.8 months was observed in a single arm phase II trial of patients with extra-pancreatic NET treated with ziv-aflibecept.

The objective response rate by RECIST 1.1 criteria to ramucirumab was low at 5 (95% CI, 0.6%-15.8%). However, this is consistent with relatively low response rates that have been observed with other targeted agents including everolimus and tyrosine kinase inhibitors that can improve progression-free survival in patients with advanced NET by slowing disease progression and achieving stable disease without an objective response. Multiple limitations of RECIST criteria NEN have been reported which include that RECIST criteria does not provide a measure of non-dimensional tumor response like necrosis, or factor in fibrotic responses to mesenteric tumor burden, nor factor functional changes of tumors.^14^ In other indolent diseases, criteria has been developed to measure tumor enhancement which permits the assessment of active and necrotic tumor tissue. In NEN, Choi criteria initially applied to GIST and liver metastases of colon cancer, correlated with time to progression in a small retrospective series of pancreatic NEN patients’ treatment with sunitinib or everolimus. Unfortunately, this criterion carries limitations regarding the reproducibility of tumor size changes.^15^ A second criteria, Chun criteria, have proven to be relevant in assessing metastatic colorectal cancer involving the liver when a patient’s regimens include bevacizumab.^16^ These efforts suggest that further evaluation and application in prospective NEN trials is needed.

Adverse events that were observed in this study are consistent with what has been reported with single-agent ramucirumab in other trials.^17^ Notably, the incidence of hypertension in patients with NETs who receive a VEGF-targeted therapy is higher than in patients with other tumors. The mechanism has not been fully elucidated but likely involves the vascular nature of NETs when compared to other tumor types, vasoactive peptides secreted by NETs, and potentially the concurrent use of somatostatin analogs which can also be associated with hypertension.^18^ In this trial, almost half of the patients had hypertension prior to enrollment. Fifteen (35%) patients developed grade 3 hypertension on ramucirumab, including 5 patients who did not have hypertension at baseline. Because of the relatively high rates of hypertension that develop, close monitoring of blood pressure and a proactive plan for managing hypertension is needed in patients with NET who are treated with VEGF pathway inhibitors.

The limitations of this study deserve comment. This was a single-arm trial that included a small number of patients which limits our ability to assess the benefits and limitations of ramucirumab in extra-pancreatic NET. Although the response rate to treatment was modest, the impact on median PFS suggests that the radiographic response rate measured by RECIST may not reflect the antiproliferative effect of targeted agents that can slow disease progression in diseases like NET.

In summary, treatment with ramucirumab was associated with encouraging efficacy in patients with advanced, progressive extra-pancreatic NET. The median PFS duration of 14.2 months is consistent with what has been observed with other VEGF pathway inhibitors that have demonstrated activity in NET. There are ongoing efforts evaluating the role of ramucirumab in patients with NET, including the phase II RamuNET trial combining ramucirumab with dacarbazine in the treatment of progressive well-differentiated metastatic pancreatic neuroendocrine tumors.^19^ The results of our study and the ongoing efforts highlight a potential role for ramucirumab in the treatment of NET. Continued investigation of ramucirumab and other inhibitors of the VEGF pathway for the treatment of NET is warranted.

## Data Availability

The data that support the findings of this study are available from the corresponding author upon reasonable request.
